# Individual trajectory-based care for COPD: getting closer, but not there yet

**DOI:** 10.1183/23120541.00451-2021

**Published:** 2021-12-13

**Authors:** Nicolas Roche, Philippe Devillier, Patrick Berger, Arnaud Bourdin, Daniel Dusser, Jean-François Muir, Yan Martinat, Philippe Terrioux, Bruno Housset

**Affiliations:** 1 Pneumologie, Hôpital Cochin, AP-HP. Centre - Université de Paris, Institut Cochin (UMR1016), Paris, France; 2 UPRES EA 220, Université Versailles Saint-Quentin, Pôle des Maladies des Voies Respiratoires, Hôpital Foch, Suresnes, France; 3 Service d'exploration fonctionnelle respiratoire, CIC 1401, CHU de Bordeaux, Pessac, France; 4 Département de Pneumologie et Addictologie, Centre Hospitalier Universitaire de Montpellier, Montpellier, France; 5 Service de Pneumologie, Oncologie Thoracique et Soins Intensifs Respiratoires, Centre Hospitalier Universitaire de Rouen, Rouen, France; 6 Pneumologie, Centre Médical Parot, Lyon, France; 7 Pneumologie, Meaux, France; 8 Service de Pneumologie, Hôpital Intercommunal de Créteil, Créteil, France

## Abstract

Chronic obstructive pulmonary disease (COPD) is a main cause of death due to interplaying factors, including comorbidities that interfere with symptoms and response to therapy. It is now admitted that COPD management should be based on clinical symptoms and health status and should consider the heterogeneity of patients’ phenotypes and treatable traits. This precision medicine approach involves a regular assessment of the patient's status and of the expected benefits and risks of therapy. The cornerstone of COPD pharmacological therapy is inhaled long-acting bronchodilation. In patients with persistent or worsened symptoms, factors likely to interfere with treatment efficacy include the patient's non-adherence to therapy, treatment preference, inhaler misuse and/or comorbidities, which should be systematically investigated before escalation is considered. Several comorbidities are known to impact symptoms, physical and social activity and lung function. The possible long-term side-effects of inhaled corticosteroids contrasting with their over-prescription in COPD patients justify the regular assessment of their benefits and risks, and de-escalation under close monitoring after a sufficient period of stability is to be considered. While commonly used in clinical trials, the relevance of routine blood eosinophil counts to guide therapy adjustment is not fully clear. Patients’ characteristics, which define phenotypes and treatable traits and thus guide therapy, often change during life, forming the basis of the concept of clinical trajectory. The application of individual trajectory-based management of COPD in clinical practice therefore implies that the benefit:risk ratio is regularly reviewed according to the evolution of the patient's traits over time to allow optimised therapy adjustments.

## Abstract 2

**In the current era of precision medicine, COPD management needs to be regularly adjusted based on the patient's personal clinical trajectory, *i.e.* the evolution of their treatable traits over time.** https://bit.ly/3kxVgC7

## Introduction

Chronic obstructive pulmonary disease (COPD) remains one of the main causes of death in Western countries, just behind cardiovascular diseases [1]. In addition to a possible insufficient implementation of guidelines in clinical practice [2], several interplaying factors can explain this situation. First, COPD is often under-diagnosed, so its management may start too late. Second, no specific marker-associated target therapy is available to date, so the management of COPD is still based on therapies aimed at reducing symptoms but with no firmly established effect on the natural evolution of the disease [1]. Third, long-term adherence of patients to pharmacologic and non-pharmacologic therapies is relatively low [3, 4], putting them in a negative spiral of inactivity, reduced quality of life and increased risk of early mortality. Fourth, most patients suffer from other morbidities related to genetic and/or environmental factors or ageing [5–9], which may impact clinical outcomes, inhaler technique and the benefits of COPD-oriented therapies. All these parameters are expected to interfere in a manner that is both specific to each patient and progressive with time according to the patient's comorbidities and phenotype.

COPD heterogeneity and its relation to COPD outcomes have been extensively explored over the past decade. For instance, data from the ECLIPSE cohort, including patients from 12 countries, showed that the prevalence and time of onset of comorbidities were independent of the Global Initiative for Chronic Obstructive Lung Disease (GOLD) stage, and that the severity of airflow limitation was poorly related to the degree of breathlessness, health status or the presence of comorbidity [10]. Furthermore, around 40% of subjects with severe airflow obstruction did not report symptoms. The multifactorial discrepancy between airflow limitation and symptoms led several groups to define clinical COPD phenotypes according to respiratory characteristics and comorbidities [11]. At the same time, an update of the GOLD document proposed to separate spirometric 1–4 grades from clinically defined “ABCD” groups. This major change suggests that, while airway obstruction remains crucial to establish the diagnosis of COPD and define its severity, initial therapy, monitoring and follow-up have to be primarily based on clinical outcomes, health status and factors interfering with symptoms and treatment effects [1]. Thus, the “one size fits all” approach is no longer recommended and should be supplanted by a tailored follow-up. This aligns well with the current “P4” concept of precision (personalised, preventive, predictive and participative) medicine [12].

Accordingly, follow-up of patients with COPD should take into account the combination of specific phenotypes, comorbidities, patients’ cognitive and functional capacities over time, treatment preferences, and expectations [13]. The ultimate goal of this approach is to estimate the likelihood of improving clinical outcomes with a given therapeutic intervention while minimizing the risks associated with unjustified prescription or maintenance of drugs known to induce adverse events [12, 14, 15]. This concept results in a strategy specific to each patient, targeting pulmonary, extra-pulmonary and behavioural treatable traits. Most importantly, patients’ characteristics should not be considered as static as they constantly evolve over time, underlining the need for a longitudinal vision of individual trajectories. Once it is admitted that the precision medicine approach is useful and has to be considered longitudinally rather than cross-sectionally in order to capture the trajectories of treatable traits over time, the subsequent challenge is the feasibility of implementation in routine clinical practice.

This paper aims to discuss the importance of individualizing patients’ trajectories when caring for patients with COPD, and the applicability of this approach in a “real life” setting on the basis of key questions to be addressed at the time of the visit. Regarding pharmacological maintenance therapy, we will focus on inhaled corticosteroids (ICS) since these drugs represent the most common treatment with a potential for individualisation based on patients’ characteristics.

## Lung function trajectories in COPD

One patient's individual COPD trajectory refers to the evolution over time of COPD-associated features and outcomes resulting from interacting genetic and environmental factors, modulated by behavioural influences [1]. These factors determine the individual dynamics of airflow limitation over the patient's lifetime, which are characterised by great inter-individual heterogeneity [16]. It is now admitted that two lung function trajectory phenotypes can lead to COPD, *i.e.* a rapid decline in patients with normal forced expiratory volume in 1 s (FEV_1_) value in early adulthood, and an abnormal lung growth and development in childhood but with a rather normal kinetics of decline thereafter [16]. Notably, as reviewed by Agusti and Faner [17], patients with low FEV_1_ values (≤80% of pred. value) in early adulthood are more likely to experience comorbidities and premature mortality.

The early-COPD phenotype has been defined by an FEV_1_/forced vital capacity (FVC) ratio below the normal limit coupled with computed tomography abnormalities (including visual emphysema) and/or an accelerated FEV_1_ decline (≥60 mL·year^–1^) [18]. According to the recent analysis of a Danish contemporary population-based cohort, it could be present in 15% of people aged under 50 years old and with a tobacco consumption of at least 10 pack years [19]. During a 14-year follow-up and after multivariable adjustment, individuals with early COPD were found to display significant increased risks of acute obstructive lung disease, pneumonia-related hospitalisation and early death. These findings suggest that early intervention could improve long-term outcomes, although this remains to be demonstrated. Thus, studies aiming to identify patients with early lung function decline are critical to improve prevention and early management.

## The weight of COPD comorbidities in individual patients’ trajectories

Observational studies suggest that up to 90% of COPD patients have at least one associated morbidity, and that up to half have three or more chronic comorbidities [20, 21]. The mean number of comorbidities is around four and increases with age [20] throughout the patient's trajectory. The most prevalent comorbidities are linked to cardiac (arterial hypertension, arrhythmias, ischaemic heart disease, chronic heart failure) or metabolic (lipid disorders, diabetes) conditions [8]. Regarding gender, osteoporosis and anxiety are more prevalent in women, while cardiovascular conditions or obstructive sleep apnoea are more prevalent in men [20, 22]. A variety of these comorbidities contribute to determine individual patients’ trajectories in that they are associated with impaired outcomes and prognosis in patients with COPD. For instance, the presence of heart failure was shown to increase the risk of death by 30–90% depending on the study [8]. Interestingly, hyperinflation (that is associated with COPD prognosis) can be mechanically involved in the pathogenesis of heart failure, especially in patients with emphysema-predominant COPD [23].

Osteoporosis is prevalent in COPD patients (15–20% of patients depending on the study), but with a less strong association with mortality [6, 9]. Yet, the presence of osteoporosis may be associated with thoracic vertebral compression fractures leading to kyphosis worsening, an increased dyspnoea and a decrease in vital capacity [8] and may consequently alter lung function [24]. In COPD patients with arthritis, joint stiffness and muscle weakness are common symptoms expected to limit physical activity [25].

**FIGURE 1:**
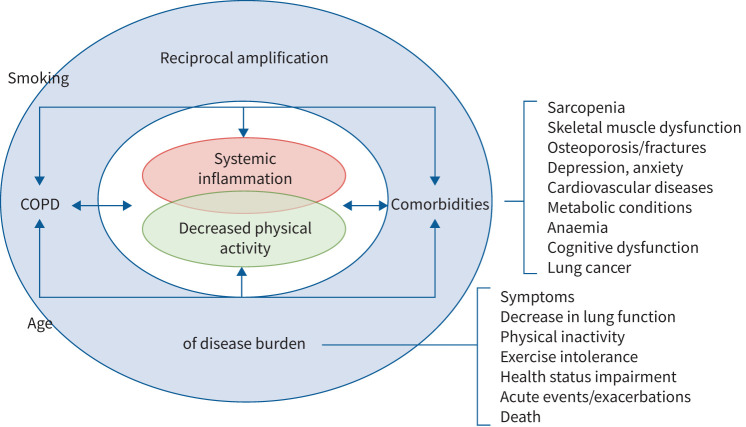
The complex interplay between chronic obstructive pulmonary disease (COPD) and its comorbidities as determinants of disease burden throughout patients’ trajectories.

Sarcopenia also represents an underestimated COPD comorbidity, being present in 25–30% of patients [8]. The reduced activity of COPD patients [3], often coupled with a deficit of nutrients (protein and vitamin D), induces a decrease in muscle mass resulting in a vicious circle of deconditioning and deterioration of exercise capacity [26]. Regarding mental health, data from the observational ECLIPSE study showed that depression would be twice as prevalent in subjects with COPD when compared with smokers without COPD (26% *versus* 12%), and that undetected and untreated depressive symptoms may increase physical disability and morbidity [27, 28]. Overall, most patients with COPD can be considered as multimorbid, with comorbidities influencing outcomes and mortality through a negative spiral (figure 1).

Regarding acute comorbidities, community-acquired pneumococcal pneumonia is seven times more frequent in COPD adults than in healthy persons of the same age and significantly enhances the risk of hospitalisation and death [29]. A cluster analysis of COPD patients showed that the risk of pneumonia was higher in patients with severe obstruction (FEV_1_/FVC <46% predicted), low body mass index (<19 kg·m^–2^), presenting with multiple comorbidities or using psychoanaleptics [30]. As discussed below, the risk of pneumonia in patients with COPD is significantly increased by the use of ICS.

## COPD management cycle: from guidelines to practical issues

The recommended management cycle highlighted in the GOLD document implies the re-assessment of the benefit:risk ratio of current therapy throughout the clinical trajectory of the patient. The purpose of this approach is to identify all factors that could have interfered with benefits and risks since the previous visit [1]. Making accurate decisions within the limited time of the visit is a true challenge that requires major issues to be addressed, including searching for possible causes of symptom persistence or worsening, questioning the need for drugs with possible side effects, and discussing the benefits and risks of de-escalation. This specifically applies to ICS, which have the potential to induce adverse effects in the long term while not being effective in all patient subtypes. However, the use of these agents remains largely inappropriate in real-life studies [31], which may improve if clinicians consider more thoroughly the concept of clinical trajectory and use it to adapt treatment strategies.

### Pharmacological therapy in COPD: a special focus on the good candidates for ICS

The rationale for focusing on ICS is the debate surrounding their benefit:risk ratio, especially considering the potential for infectious and systemic side effects. An important consideration is that the determinants of the benefit:risk ratio are likely to change over time in a given patient, which supports integrating their assessment throughout the patient's trajectory.

Some recommendations or propositions for ICS use in COPD are summarised in table 1 [1, 32–36]. Overall, as soon as dyspnoea becomes persistent, COPD patients should be preferentially given long-acting bronchodilators, including muscarinic antagonists (LAMA) and/or long-acting beta2 agonists (LABA); LAMA being preferred in patients with recurrent exacerbations and in whom monotherapy is considered [1]. ICS/LABA combination may be considered in situations suggesting steroid responsiveness, including high blood eosinophil counts and/or the presence of features of asthma [1, 35, 36].

**TABLE 1:** Recommendations for inhaled corticosteroid use or withdrawal in chronic obstructive pulmonary disease according to several international and national guidelines

**Guideline**	**Initiation**	**Escalation**	**De-escalation** ^#^
**GOLD 2021** [1]	In most cases (GOLD A–C and most D): LAMA or LABA or LAMA/LABA.	LAMA or LABA to ICS/LABA: eosinophils ≥300·µL^−1^ or and two moderate exacerbations/one hospitalisation.	Pneumonia, inappropriate initial indication and/or lack of response to ICS. De-escalation: close monitoring if eosinophils ≥300·µL^−1^.
	ICS/LABA option: if dyspnoea and exacerbations (GOLD D) and eosinophil counts ≥300·µL^−1^.	LAMA/LABA to triple therapy: exacerbations and eosinophils ≥100·µL^−1^.	
**ATS 2020** [32]	If dyspnoea: LAMA/LABA.	LAMA/LABA to triple therapy: ≥1 exacerbation in the past year.	No exacerbations in the past year.
	ICS/LABA option: ≥1 exacerbation in the past year, if eosinophil counts ≥150·µL^−1^ (2%).		
**ERS 2020** [33]	No specific guidelines regarding ICS.		Blood eosinophils <300·μL^−1^ and no frequent exacerbations.
**SPLF 2021** [34]	Preferred option: LAMA^¶^ or LABA.	LAMA or LABA to ICS/LABA: exacerbations and no dyspnoea (mMRC <2), eosinophil count (>300·µL^−1^) to be considered as a secondary criterium.	ICS-associated adverse events, eosinophils <300·µL^−1^ or no exacerbation in the past year.
	ICS/LABA: not recommended.	LAMA/LABA to triple therapy: dyspnoea and/or ≥1 severe or ≥2 moderate exacerbations in the past year.	
**NICE 2019** [35]	Preferred option: LAMA/LABA.	LAMA/LABA to triple therapy: daily symptoms that adversely impact quality of life or one severe or two moderate exacerbations within a year.	Symptoms not improved after 3 months on triple therapy.
	ICS/LABA: if asthmatic features or features suggesting steroid responsiveness^+^.		
**CTSCPG 2019** [36]	LAMA or LABA if low risk of AECOPD^§^.	LAMA or LABA to ICS/LABA: if concomitant asthma.	No improvement in dyspnoea, exercise tolerance or health status, and no history of frequent and/or severe AECOPD improved by triple therapy.
	ICS/LABA: ≥1 exacerbation in the past year and eosinophils ≥300·µL^−1^.	LAMA/LABA to triple therapy: persistent. dyspnoea and poor health status in the last year.	

^#^De-escalation: triple therapy (LAMA/LABA/ICS) to LAMA/LABA. ^¶^Preferred option in patients with exacerbations. ^+^Higher blood eosinophil count, substantial variation in forced expiratory volume in 1 s (FEV_1_) over time (≥400 mL) or substantial diurnal variation in peak expiratory flow (≥20%). ^§^Low *versus* high risks of AECOPD: ≥1 moderate AECOPD *versus* ≥2 moderate AECOPD or ≥1 severe AECOPD (hospitalisation) in the last year.

AOCOPD: acute exacerbation of chronic obstructive pulmonary disease; ATS: American Thoracic Society; COPD: chronic obstructive pulmonary disease; CSI: corticosteroid inhaler; CTSCPG: Canadian Thoracic Society clinical practice guideline; ERS: European Respiratory Society; GOLD: Global Initiative for Chronic Obstructive Lung Disease; ICS: inhaled corticosteroid; LAMA: long-acting bronchodilators, including muscarinic antagonists; LABA: long-acting beta2 agonists; mMRC: modified Medical Research Council; NICE: National Institute for Heath and Care Excellence; SPLF: French Respiratory Society.

Despite common features and traits, there are marked clinical, pathophysiological, morphological and evolutionary differences between COPD and asthma that need to be systematically appraised during the diagnostic process. Importantly, physicians need to question the original diagnosis whenever the situation worsens, since asthma and COPD may coexist or present with initially misleading features. Indeed, the individual trajectory of some patients is characterised by diagnostic changes or associations. While eosinophils represent the main effector cell in asthma [37], airway inflammation in COPD typically involves macrophages, neutrophils and lymphocytes [38, 39]. As a consequence, while asthma management is based on ICS [40], bronchodilation is the cornerstone of COPD therapy to reduce or prevent symptoms and exacerbations of COPD [1]. However, some COPD patients may exhibit elevated levels of eosinophils in the airways, although this feature is not always stable over time [41]. Furthermore, several factors including bacterial/viral infections and comorbidities are expected to increase the risk of exacerbation or to have a negative impact on their frequency [42]. Thus, the term “exacerbation” refers to different causes (bacterial, viral, inflammatory, eosinophilic) [43]. Considering this heterogeneity of exacerbation mechanisms is likely crucial to provide specific treatments and prevention strategies.

While possible, the coexistence of asthma in COPD patients should not be overestimated [44]. The prevalence of asthma and COPD overlap (ACO)/asthma+COPD is highly dependent on the definition used, and recent data from a prospective Korean cohort clearly indicate that the identification of ACO on the basis of questionnaires would range between 3% and 30% depending on the criteria used [45]. The analysis showed that the only factor associated with a decrease in ACO exacerbation after ICS use was a blood eosinophil count of ≥300 cells·μL^–1^ irrespective of asthma history, suggesting the need to use ICS based on this treatable trait rather than a disease label. Accordingly, the most recent GOLD document no longer refers to ACO as a particular entity, emphasizing that COPD and asthma are mostly different disorders to be managed accordingly [1]. In practice, prescribing ICS simply because of some putative past asthma history (or post-bronchodilator reversibility) is not desirable as it can lead to unjustified corticosteroid-based therapy and potential ICS-associated adverse events [46].

The most widely described ICS adverse effect in COPD patients is pneumonia, which has been documented in both clinical randomised and cohort-based studies. The *post hoc* analysis of the TOwards a Revolution in COPD Health (TORCH) clinical study comparing fluticasone/salmeterol to salmeterol alone (or placebo), showed a rate of pneumonia after adjustment for time on treatment of 80 *versus* 50 events per 1,000 treatment years, respectively [47]. A Canadian cohort study based on 160,000 patients with COPD showed that the risk of severe pneumonia with current use of ICS was increased by nearly 70%, and gradually declined after discontinuation [48]. Regarding patients at risk of ICS-induced pneumonia, a double-blind clinical trial showed that the twofold increase in the risk of radiographically confirmed pneumonia observed at 1 year was associated with current smoking, having prior pneumonia, a body mass index less than 25 kg·m^–2^ and severe airflow limitation [49]. ICS have also been suspected to induce or aggravate other COPD-associated comorbidities including diabetes mellitus, osteoporosis and related bone fractures, whose risk of onset in COPD patients correlates with ICS cumulative doses [50]. Although these systemic ICS side effects have been mostly demonstrated in observational studies, some evidence from randomised clinical trials does exist, at least for fractures (although with some inconsistency) and skin bruising [51, 52], which definitely supports the occurrence of clinically relevant systemic exposure [53].

When the response to an initial treatment with an LAMA or an LABA is not sufficient, escalation may consist of a combination of LAMA/LABA or ICS/LABA. According to the GOLD document, the latter is a possible option in patients with both a high risk of exacerbations (≥2 moderate acute exacerbations of COPD (AECOPD) or ≥1 severe exacerbation requiring hospitalisation in the last year), and high blood eosinophil counts [1]. Yet, the effectiveness of bronchodilators on exacerbation risk suggests that in patients with both a risk of exacerbation and a high dyspnoea grade, a dual bronchodilator therapy may be more appropriate in terms of benefits and risks, as proposed by the French Respiratory Society [34], and suggested in the GOLD document.

Current international guidelines include the possibility of an escalation from dual therapy to LAMA/LABA/ICS triple therapy in patients remaining symptomatic, after having ruled out factors that could interfere with the efficacy of the current therapy (see below) and after having evaluated the risks of adding ICS [1]. The triple therapy option is based on recent trials including patients enriched for increased respiratory symptoms and a history of frequent or severe exacerbations [54–57]. The difference in favour of the triple therapy *versus* bronchodilators, only observed on the rate of exacerbations, was in proportion with blood eosinophil counts [58], and a threshold of 100·µL^−1^ has been retained in the GOLD document when considering escalation from LAMA/LABA to triple therapy [1].

Overall, the relevance of blood eosinophil counts for prediction of exacerbation risk and treatment response in routine clinical practice remains to be further explored [59, 60]. On the basis of data from randomised trials and observational studies, low values (<150·µL^−1^ or <2%) predict a low likelihood of a response to ICS, while high counts (≥300·µL^−1^ or ≥4%) suggest a greater response to ICS. In that context, one may consider that subcategorizations of patients according to blood eosinophil levels contribute to tailored therapy and may help clinical decision-making when escalation or de-escalation is considered [61]. Importantly, these results mostly apply to populations enriched with patients with a history of frequent exacerbations, who may not be representative of patients followed in primary care [62]. While eosinophil count remains a major parameter in the context of clinical trials to select patients susceptible to respond to ICS [63], or to better define inflammatory endotypes, particularly in studies aimed at assessing new strategies with biologics, its relevance in routine clinical practice at an individual level remains to be documented. In addition, it must be kept in mind that a rise in eosinophil counts may be induced by comorbidities (*e.g.* allergic, rheumatologic, infectious, neoplastic and other disorders) [64]. Thus, one may consider that clinical outcomes should prevail as guides to therapy adjustment, and that eosinophil counts may help to make decisions in some specific clinical situations. In this respect and as highlighted in the guidelines of the American Thoracic Society (ATS), randomised clinical trials should stratify patients by eosinophil level and exacerbation risk in order to define the most predictive threshold values for various patient categories [32]. In addition, it must be kept in mind that COPD therapeutic studies published in recent years differ somehow in terms of patient characteristics, with some allowing patients with a past history of asthma while others set thresholds of lung function reversibility or eosinophil count. These variations may contribute to explaining differences in the respective effects of strategies based on bronchodilator-only or ICS-containing combinations.

Of note, several questions on pharmacological treatment adaptation and the role of ICS in patients with COPD remain unanswered due to the lack of adequate studies. For instance, the respective benefit:risk ratio of the various available ICS/LABA combinations at various dosages have not been directly compared. In patients with exacerbations on dual bronchodilation, adding azithromycin (or mucoregulators) has not been compared to add-on ICS. Such comparative studies would help in making more informed decisions but are clearly difficult to conduct, especially considering the relatively limited magnitude of treatment effects in COPD (as opposed to asthma), making it necessary to include large populations followed over at least 1 year.

If the current guidelines were applied, one could expect a limited level of ICS prescription in COPD patients. Indeed, as mentioned above, the rate of coexistence of COPD and asthma is low, and only a minor proportion of COPD patients can be actually classified as frequent exacerbators [62, 65]. For instance, data from the 3-year follow-up of the prospective SPIROMICS cohort showed that only 2.1% of COPD patients had repeatedly two or more AECOPDs in each year of follow-up [62]. This observation is consistent with an analysis of the UK primary care electronic healthcare records that indicates that more than 80% of COPD patients would be graded with no or rare moderate exacerbations [66]. Yet, data from the same database suggest that over two-thirds of COPD patients are prescribed ICS-containing therapy – in particular triple therapy – in routine clinical practice, which clearly indicates that ICS-containing regimens are commonly but often inappropriately prescribed in COPD patients [67]. An over-prescription of ICS in COPD, including treatment initiation, has also been reported in other European observational studies [68, 69]. In the USA, a retrospective study based on medical and pharmacy data suggests that 10% of COPD patients would be prescribed ICS-containing triple therapy without prior bronchodilator use or exacerbation history [70]. Poor adherence to guidelines is a worldwide issue, as supported by the results of a Korean prospective study that revealed that more than half of inappropriate treatments were observed in patients classified GOLD B (according to GOLD 2017 guidelines), with 44% having been prescribed a triple therapy [71]. This underlines the need to re-evaluate treatment indications regularly during the patient's trajectory, considering that the “frequent exacerbator” treatable trait may vary spontaneously over time.

### Non-appropriate response to therapy: what may be hidden

Since many interacting factors related to the patient's behaviour and/or comorbidities can interfere with COPD symptoms and response to therapy, no escalation should be considered before having ruled out possible causes of “insufficient” therapy [1]. In such a situation, three key questions should be addressed:

#### Are all appropriate non-pharmacological interventions implemented?

The importance of assessing and stimulating physical activity in patients with COPD has been clearly stated, in particular by the European Respiratory Society (ERS) on the basis of large studies showing an inverse relationship between physical activity levels and the magnitude of lung function decline, or the risk of hospitalisation and mortality [72]. While the benefits of respiratory rehabilitation (RR) on exercise capacity, dyspnoea, fatigue and health-related quality of life are clearly demonstrated [73], as well as the effects of pharmacotherapy on exercise capacity or endurance [74, 75], only a few randomised studies addressed the effects of combining RR and long-acting bronchodilators. Treatment with tiotropium has been shown to significantly amplify the benefits of RR on endurance time when compared with rehabilitation alone [76]. More recently, the randomised PHYSACTO study assessed the benefits of the combination tiotropium/olodaterol on exercise endurance time in patients participating in a self-management behaviour-modification programme, in comparison with the association with RR [77]. The results showed that the significant effect of tiotropium/olodaterol on exercise endurance time was amplified by RR.

Other non-pharmacological interventions have to be systematically re-checked before considering therapy adjustment. Regarding smoking status, “real-life” data suggest that one-third of COPD patients are still active smokers [78], so cessation encouragement remains a major issue. Nicotine replacement therapy and nicotine-receptor agonists like varenicline have been shown to increase cessation rates in patients with COPD [79]. Regarding prevention of lower respiratory tract infections, despite clear evidence and recommendations supporting influenza and pneumococcal vaccinations as key interventions in COPD management, they remain insufficiently applied in real-life settings [80].

Checking the implementation of these interventions whenever the patient's trajectory is characterised by persistent or worsened symptoms should be compulsory before any adjustment of therapy.

#### Is adherence optimal?

Poor adherence to pharmacological therapy is common in patients suffering from chronic diseases, including COPD, particularly when the benefit of treatment is not immediately perceived [81]. Non-adherence may be intentional (no benefit perceived, fear of adverse events), unintentional (misunderstanding, cognitive decline) or irregular (neglect, omission) [82]. Furthermore, the extensive number of medications associated with comorbidities may decrease adherence. A study assessing adherence in more than 14,000 US COPD patients showed that the mean proportion of days covered for COPD medications was 47%, whereas it was around 70% for antihypertensives or antihyperglycaemics [4]. Multivariate analyses showed that non-adherence to other drugs prescribed for comorbidities was independently associated with increased odds of non-adherence to COPD medications.

A real-world analysis including 1,433 COPD patients followed in France, Germany, Italy, Spain and the UK showed a direct relationship between inhaler satisfaction and treatment adherence [83]. Misuse of inhalers is highly frequent in patients with COPD and is associated with impaired clinical outcomes, including an increased risk of exacerbation [84, 85]. It may also be aggravated by ageing and some comorbidities, as detailed in the next section. In the future, the use of smart inhalers may help decrease the rate of inhaler misuse, which has disappointingly not improved over the last decades [86].

As highlighted in the GOLD document, adherence to therapies and inhaler technique should be systematically re-assessed throughout the patient's trajectory before concluding that the current therapy is insufficient [1].

#### To what extent could comorbidities or ageing interfere with symptoms and/or treatment efficacy?

Respiratory infection, including bronchiectasis, tuberculosis or viral infections, as well as cardiovascular comorbidities (*e.g.* heart failure, atrial fibrillation) may directly impact or mimic COPD-associated symptoms. Impaired outcomes related to heart disease may be frequent due to common risk factors (tobacco, ageing, pollution, decreased physical activity), comorbidities (hypertension, hyperlipidaemia, diabetes, infections) and pathophysiological processes (inflammation, oxidative stress) [6]. Thus, in a context of symptom worsening, a cardiovascular work-up may be of help.

Further, comorbidities like osteoporosis or sarcopenia may alter lung function as the result of kyphosis-associated decrease in vital capacity or muscle weakness [8, 87]. A study in consecutive ambulatory patients with COPD undergoing pulmonary function testing showed a significant correlation between inspiratory capacity and peak inspiratory flow rate [24]. Thus, vertebral fractures and kyphoscoliosis could contribute to impairing not only lung function but also the ability to properly use dry powder inhalers (DPIs). Indeed, DPIs require inspiratory flows greater than 30 mL·min^−1^ and up to 60 mL·min^−1^ for some DPIs [88, 89], while pressurised metered-dose inhalers and soft mist inhalers require a slow and steady inhalation with an inspiratory flow of 15–30 mL·min^−1^ [87]. A decrease in peak inspiratory flow has also been linked to ageing, independent of the severity of airflow obstruction [90]. Ageing is a well-established situation associated with functional and cognitive capacity loss known to jeopardise the correct and efficient use of the inhaler, depending on both the patient's capacity and the inhaler characteristics [91]. In addition to a reduction in inspiratory performance, factors impacting inhaler use in the elderly may include tremors, difficult hand–eye coordination and/or loss of dexterity and strength.

Algorithms have been developed to help physicians choose the right inhaler relative to the patient's current cognitive and functional capacity that may have declined since the previous visit [88, 92].

### Therapeutic de-escalation: why, when, how?

Importantly, intensification should not be considered as the only way of adapting treatment throughout COPD patients’ trajectories. A clear and consensual situation of recommended de-escalation is when therapy was started without a clear indication, like the use of an ICS in a patient with no history of exacerbations [1, 33, 36, 50] (table 1). In the GOLD document, de-escalation exclusively consists of ICS withdrawal and has to be considered in the absence of clinical benefit of ICS, in patients who return with resolution of symptoms and/or when side effects occur, regardless of eosinophilia [1]. However, in a recent clinical practice guideline on de-escalation in COPD, the ERS recommends not withdrawing ICS in patients with blood eosinophil counts of 300·µL^−1^ or more, whatever the history of exacerbation [33]. As discussed above, whether and how eosinophil counts must be considered before treatment adjustment is not fully consensual.

The feasibility of de-escalation in patients on triple therapy is mostly based on two studies with different designs. The WISDOM study assessed the impact of progressive withdrawal of fluticasone in patients with a history of at least one documented exacerbation in the 12 months before screening and who had been on the triple therapy tiotropium, salmeterol and fluticasone for at least the 6-week run-in period [93]. The results at 52 weeks showed that the probability of experiencing moderate or severe exacerbation was not higher after ICS withdrawal than in patients maintained on triple therapy, whatever the GOLD category at baseline. ICS withdrawal was associated with a mean decrease in FEV_1_ of 43 mL at 52 weeks. A *post hoc* analysis showed that high blood eosinophil counts at screening (>300·µL^−1^) were associated with exacerbation rates reported after complete ICS withdrawal in patients with severe to very severe COPD and a history of exacerbations [94]. The SUNSET study compared the continuation of a triple therapy, including tiotropium plus a combination salmeterol/fluticasone propionate, with a dual therapy indacaterol/glycopyrronium in patients who had stable COPD for at least 6 months and who were not frequent exacerbators [95]. Like in the WISDOM study, the annualised rate of moderate or severe COPD exacerbations was not higher in patients on dual therapy, which supports the possibility of an abrupt ICS withdrawal. Again, a sub-group analysis according to blood eosinophil counts showed that the equivalence was no longer observed with eosinophils ≥300·µL^−1^, a situation associated with a higher risk of exacerbation with dual therapy.

While the rationale and feasibility of ICS withdrawal regarding benefits and risks are documented, the appropriate timing is not clear. Analysis of Danish nationwide healthcare registry data including patients with different trajectories showed a high rate of persistence of ICS-containing therapy despite improvement in exacerbation status at 4 years, which made the authors emphasise the need for developing and implementing recommendations for de-escalation strategies in clinical practice [64]. The ATS proposes a duration of 1 year without exacerbations before considering ICS withdrawal [32]. Regarding monitoring following de-escalation, the GOLD document indicates that de-escalation should be undertaken under close medical supervision [1].

## Precision medicine in COPD: yes, we can

**FIGURE 2:**
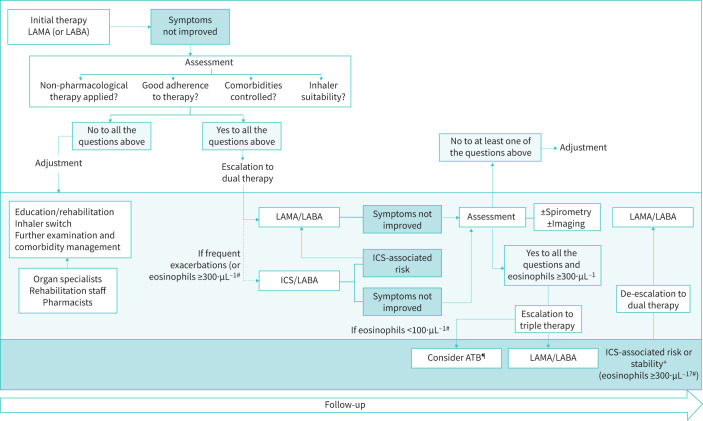
Representation of the trajectory-based approach to chronic obstructive pulmonary disease (COPD) management. The two major issues to be considered are the worsening-associated factors to be sought and identified before escalating, and the relevance of de-escalation in patients on triple therapy at risk of inhaled corticosteroid-associated adverse effects or with no benefits. Taking into consideration blood eosinophil counts is mentioned in the current guidelines but their individual relevance in routine practice is not fully clarified. ^#^Eosinophil counts to be considered according to an individual approach and particularly in borderline situations. ^¶^ATB: macrolide-based therapy. ^+^Stability remains to be defined: at least 1 year according to the ATS guidelines, or 2 years according to the definition of frequent exacerbations.

The management cycle of COPD based on individual patients’ traits and trajectories may appear as a new paradigm and a new challenge for physicians. Yet, it just refers to the need for determining the most appropriate regimen adjustment according to the course of the patient's traits, health status, lifestyle and treatment-associated risks over time (figure 2).

The use of precision medicine in COPD requires the recognition and assessment of the patient's multilevel, specific and dynamic treatable traits [14]. In practice, this personalised approach implies that physicians are able to take into consideration all the factors guiding therapy within the (short) duration of a medical visit. The first key question to be addressed is the clinical “control” of the disease according to the goals defined during the previous visit and the changes having occurred since then. Several criteria may be considered to determine whether the disease is controlled or not, including health status (assessed using, for instance, COPD Assessment Test (CAT) score or clinical COPD questionnaire), dyspnoea grade, and number and severity of exacerbations. In an international prospective observational study in primary care or specialised centres, and comprising five evaluation points, the percentage of control ranged from a minimum of 13% with the most stringent criteria to a maximum of 32% when only the CAT score was used to assess clinical impact and stability [96]. Soler-Cataluña *et al.* [97] recently proposed a two-dimensional composite modified control criterium (MCC) based on clinical impact (dyspnoea score, rescue medication, sputum colour, physical activity) and stability (subjective perception, exacerbations in the past 3 months). An acceptable value of the MCC was prospectively shown when using time to the first combined event. A lower predictive value was found when considering CAT score as a criterion of clinical impact (low if 0–10 or 0–16 with FEV_1_≥ or <50%, respectively) and stability (≤2 points). While being limited by the low proportion of patients with severe COPD and the absence of correlation with mortality, this study suggests that the number of patients with uncontrolled COPD might be commonly overestimated, and that simple criteria that are easy to use in clinical practice may be helpful for assessing the patient's clinical trajectory.

In practice, physicians should regularly use a checklist to assess the implementation of non-pharmacological therapies, adherence and inhaler technique and, last but not least, comorbidities that could interfere with the response to inhaled therapy. It is important to insist again here on the possible decline in cognitive and/or functional capacities over time, so that an inhaler that was initially suitable could become inadequate and put the patient in a dangerous spiral. In this context, switching to a device adapted to the patient's skills may be more appropriate than therapy escalation. If the global assessment concludes suboptimal therapy on the basis of frequent exacerbations, then the benefits and risks associated with therapeutic reinforcement should be considered.

Making decisions at an individual level implies that the global individual assessment at the time of the visit is coupled with information collected during previous visits so that treatable traits as well as therapy goals and adjustments are defined according to the personal trajectory of the patient. In this respect, local and international cohorts of COPD patients are expected to provide major information on this topic, including the nature, evolution and impact of comorbidities over time. While possibly limited by missing data inherent to studies in real-life settings, such cohorts have the advantage of allowing to include all relevant patients without any exclusion criteria. In addition, early endotyping coupled with genomic, proteomic and metabolomic studies on at-risk patients (including the younger) could be important to identify the molecular mechanisms involved in the disease trajectory [98]. Finally, one may expect a great deal from new technologies and artificial intelligence to characterise COPD subtypes based on underlying disease processes and distinct patterns of disease trajectory [99]. In the meantime, it is already the responsibility of the physician to do his/her best to maintain his/her patient in the optimal benefit:risk corridor. To move closer to precision medicine, all healthcare professionals involved in the continuum of COPD care should interact with each other and with the patient to make sure that all factors influencing his/her trajectory are considered.

In addition to the patients’ characteristics that should be used to individualise therapy, economic aspects need to be considered when making treatment choices. Their weight may vary widely between settings, but the cost effectiveness of various treatment options can be put in perspective using models such as the COPD value pyramid developed a decade ago by the London Respiratory Network with the London School of Economics [100]. Discussing this topic more extensively is outside the scope of the present manuscript.

Finally, as for any treatment strategy, implementation is key and does not depend only on scientific arguments. Proper treatment individualisation based on the patient's characteristics and trajectory requires the application of a systematic process. This process is not particularly complex, but it may be facilitated by a dedicated organisation involving, *e.g.*, reminders, decision aids, specialised allied healthcare professionals or expert clinics.

## Conclusion

The management cycle proposed in the last GOLD document implies that therapy is systematically reviewed and potentially adjusted according to the patient's individual trajectory. While such an approach may be limited by the time allocated to patient assessment in routine practice, it is essential that patients are not given (or maintained on) unjustified therapy that may increase a risk of morbi-mortality with no benefits for symptoms, health status and quality of life. Although no specific therapy can reverse the natural evolution of COPD, numerous factors related to the patient's history, comorbidities and behaviour may contribute to defining the most relevant way of adjusting management over time – provided that they are regularly reviewed. Moving towards an “individual trajectory era” should lead to stopping unjustified reflexes, so precision medicine will supplant the irrelevant “one size fits all” approach.
